# Costs of Family Building for Physicians and Medical Students

**DOI:** 10.1001/jamanetworkopen.2024.57287

**Published:** 2025-01-29

**Authors:** Morgan S. Levy, Amelia G. Kelly, Alyssa D. Brown, Roohi Jeelani, Hina Talib, Jane W. Liang, Arghavan Salles

**Affiliations:** 1 Department of Radiation Oncology, University of Kentucky College of Medicine, Lexington; 2 Department of Medical Education, University of Miami Miller School of Medicine, Miami, Florida; 3 Department of Obstetrics and Gynecology, NYU Grossman School of Medicine, New York, New York; 4 Department of Surgery, Northwestern University, Chicago, Illinois; 5 Department of Research and Education, Vios Fertility Institute, Chicago, Illinois; 6 Department of Obstetrics and Gynecology, Wayne State University School of Medicine, Detroit, Michigan; 7 Department of Pediatrics, Children’s Hospital at Montefiore, Albert Einstein College of Medicine, Bronx, New York; 8 Quantitative Sciences Unit, Department of Medicine, Stanford University School of Medicine, Palo Alto, California; 9 Clayman Institute for Gender Research, Department of Medicine, Stanford University, Palo Alto, California

## Abstract

This survey study examines the financial costs incurred by physicians and medical students in building their families.

## Introduction

Female physicians are twice as likely as the general public to face infertility.^1^ Assisted reproductive technology (ART) costs are substantial; on average, an in vitro fertilization cycle can cost more than $30 000.^2^ Health insurance often does not cover ART, gestational carriers, or adoption. To our knowledge, no previous study has characterized the financial costs incurred by physicians and medical students in building their families.

## Methods

We administered a survey, following the AAPOR reporting guidelines, to a sample of physicians and medical students on social media (Twitter [now X], Instagram, Facebook) from April to May 2021.^3^ The current analysis included participants who used ART, gestational carriers, and/or adoption. Cost was evaluated by out-of-pocket costs and insurance coverage for ART and out-of-pocket costs for gestational carrier pregnancies and adoption. All costs were self-reported, in intervals of $20 000 ranging from $0 to $20 000 up to more than $140 000 (or >$200 000 for gestational carrier and adoption) for each method of family building used. Analyses were stratified by age and level of training. Because female fertility starts decreasing around age 32 years, we compared costs between those older and younger than that age.^4^ Statistics were calculated using SPSS statistical software version 29 (IBM Corp) and χ^2^ tests, with 2-sided *P* < .05 denoting significance. Race and ethnicity were included as demographic characteristics by self-identification. The study was approved by the University of Miami Institutional Review Board. Written informed consent was obtained before participation.

## Results

Of 3810 consented participants (mean [SD] age, 40.4 [7.4] years), 3104 (81.5%) completed the survey, and 645 (20.8%) had used ART, gestational carriers, and/or adoption. The majority of participants were female (585 participants [90.7%]), heterosexual (550 participants [85.8%]), and independent practitioners having completed training (541 participants [84.0%]) (Table 1).

**Table 1.** Sociodemographic Characteristics of Participants Who Used Assisted Reproductive Technology, Adoption, and/or Gestational Carrier^a^

Characteristic	Participants, No. (%) (N = 645)^b^	*P* value
Sex^c^		
Female	585 (90.7)	.95
Male	59 (9.1)	
Prefer to describe	0	
Prefer not to answer	<5 (<0.8)	
Gender		
Woman	578 (89.6)	.19
Man	59 (9.1)	
Nonbinary	<5 (<0.8)	
Agender	<5 (<0.8)	
Gender fluid	<5 (<0.8)	
Gender queer	<5 (<0.8)	
Prefer to describe	0	
Prefer not to answer	<5 (<0.8)	
Sexual orientation		
Asexual	<5 (<0.8)	.01
Bisexual	30 (4.7)	
Demisexual	<5 (<0.8)	
Fluid	<5 (<0.8)	
Gay	20 (3.1)	
Heterosexual	550 (85.8)	
Lesbian	15 (2.3)	
Pansexual	<5 (<0.8)	
Queer	7 (1.1)	
Questioning	<5 (<0.8)	
Prefer to describe^d^	<5 (<0.8)	
Prefer not to answer	5 (0.8)	
Age, mean (SD), y	40.4 (7.4)	<.001
Title		
Medical student	23 (3.6)	<.001
Resident	40 (6.2)	
Fellow	34 (5.3)	
Independent practitioner	541 (84.0)	
Prefer to describe^d^	6 (0.9)	
Race and ethnicity		
American Indian or Alaskan Native	<5 (<0.8)	.006
Asian	128 (19.8)	
Black or African American	39 (6.0)	
Hispanic, Latinx, or Spanish Origin	<5 (<0.8)	
Middle Eastern or North African	13 (2.0)	
Multiracial^e^	35 (5.4)	
White	415 (64.3)	
Prefer to describe^d^	11 (1.7)	
Prefer not to answer	<5 (<0.8)	

^a^ The participants in this sample have used assisted reproductive technology, adoption, and/or gestational carriers to build their family. The specific Facebook groups used to recruit participants are American Medical Association Medical Students, American Medical Women’s Association, Association of Women Surgeons, Gay Men Physicians Group, LGBTQ+ Premed and Medical Students, LGBTQIA+ Med Students, Medical Student Pride Alliance, Physician Moms Group, Physician Women Warriors, and Women in Academic Medicine Leadership.

^b^ Total numbers may vary due to item nonresponse.

^c^ Another response option, which no participants in this sample selected, was intersex.

^d^ Participants who selected “prefer to describe” wrote in free-text responses.

^e^ Participants who selected more than 1 option are considered multiracial for the purpose of this study.

In total, 19.3% of all participants (597 participants) used ART (Table 2). Those with insurance coverage for ART (348 participants) incurred significantly lower out-of-pocket costs than those without insurance coverage (χ^2^_7_ = 20.475; *P* = .005). A total of 63 participants (1.9%) pursued adoption. An even smaller number of participants pursued gestational carrier pregnancies (32 participants [0.9%]). Those aged 32 years and older and in independent practice generally experienced the highest costs.

**Table 2.** Financial Burden Associated With Family Building

Variable	Participant age					Participant title								Overall, No. (%)^a^
	No. (%)^a^		χ^2^	*df*	*P* value	No. (%)^a^					χ^2^	*df*	*P* value	
	≤31 y	≥32 y				Medical student	Resident	Fellow	Independent practitioner	Prefer to describe				
Have you used ART? (n = 3063)														
Yes	40 (3.5)	549 (28.8)	296.749	1	<.001	19 (2.3)	34 (8.2)	32 (16.2)	507 (31.1)	4 (14.3)	2332.076	4	<.001	597 (19.3)
No	1115 (96.5)	1359 (71.2)				802 (97.9)	380 (91.8)	165 (83.8)	1124 (68.9)	24 (85.7)				2498 (80.7)
Out-of-pocket cost for ART, $ (n = 582)^b^														
0 to <20 000	24 (63.2)	248 (46.3)	11.649	7	.11	8 (44.4)	19 (61.3)	19 (63.3)	228 (45.8)	3 (75.0)	29.877	28	.37	277 (47.6)
20 000 to <40 000	11 (28.9)	111 (20.7)				8 (44.4)	9 (29.0)	7 (23.3)	98 (19.7)	0				122 (21.0)
40 000 to <60 000	2 (5.3)	65 (12.1)				1 (5.6)	3 (9.7)	2 (6.7)	62 (12.4)	0				69 (11.9)
60 000 to <80 000	0	38 (7.1)				0	0	0	37 (7.4)	0				38 (6.5)
80 000 to <100 000	0	30 (5.6)				1 (5.6)	0	1 (3.3)	29 (5.8)	0				31 (5.3)
100 000 to <120 000	1 (2.6)	14 (2.6)				0	0	0	15 (3.0)	0				15 (2.6)
120 000 to <140 000	0	12 (2.2)				0	0	1 (3.3)	11 (2.2)	0				12 (2.1)
>140 000	0	18 (3.4)				0	0	0	18 (3.6)	0				18 (3.1)
Total cost, $	780 000	20 220 000				460 000	610 000	720 000	19 300 000	30 000				21 240 000
ART cost covered by insurance, $ (n = 586)^b^														
No coverage	23 (60.5)	203 (38.3)	10.620	7	.16	13 (72.2)	13 (41.9)	8 (25.8)	201 (40.1)	3 (75.0)	29.573	28	.38	238 (40.6)
1 to <20 000	14 (36.8)	220 (41.5)				4 (22.2)	18 (58.1)	17 (54.8)	198 (39.5)	1 (25.0)				238 (40.6)
20 000 to <40 000	0	51 (9.6)				1 (5.6)	0	3 (9.8)	48 (9.6)	0				52 (8.9)
40 000 to <60 000	0	23 (4.3)				0	0	0	23 (4.6)	0				24 (4.1)
60 000 to <80 000	1 (2.6)	13 (2.5)				0	0	0	14 (2.8)	0				14 (2.4)
80 000 to <100 000	0	10 (1.9)				0	0	1 (3.2)	9 (1.8)	0				10 (1.7)
100 000 to <120 000	0	6 (1.1)				0	0	1 (3.2)	5 (1.0)	0				6 (1.0)
120 000 to <140 000	0	0				0	0	0	0	0				0
≥140 000	0	4 (0.8)				0	0	1 (3.2)	3 (0.6)	0				4 (0.7)
Total coverage, $	210 000	8 160 000				70 000	180 000	610 000	7 360 000	10 000				8 280 000
Cost of gestational carrier, $ (n = 32)														
0 to <20 000	2 (66.7)	7 (25.0)	7.483	9	.59	1 (50.0)	2 (66.7)	0	5 (20.8)	1 (50.0)	41.133	36	.25	9 (28.1)
20 000 to <40 000	0	1 (3.6)				0	0	0	1 (4.2)	0				1 (3.1)
40 000 to <60 000	0	2 (7.1)				0	0	1 (100.0)	0	1 (50.0)				2 (6.3)
60 000 to <80 000	0	1 (3.6)				1 (50.0)	0	0	1 (4.2)	0				2 (6.3)
80 000 to <100 000	0	3 (10.7)				0	0	0	3 (12.5)	0				3 (9.4)
100 000 to <120 000	0	5 (17.9)				0	0	0	5 (20.8)	0				5 (15.6)
120 000 to <140 000	0	3 (10.7)				0	0	0	3 (12.5)	0				3 (9.4)
140 000 to <160 000	0	1 (3.6)				0	0	0	1 (4.2)	0				1 (3.1)
160 000 to <180 000	0	0				0	0	0	0	0				0
180 000 to <200 000	1 (33.3)	1 (3.6)				0	1 (33.3)	0	1 (4.2)	0				2 (6.3)
≥200 000	0	4 (14.3)				0	0	0	4 (16.7)	0				4 (12.5)
Total cost, $	210 000	2 660 000				80 000	210 000	50 000	2 540 000	30 000				2 940 000
Cost of adoption, $ (n = 63)														
0 to <20 000	3 (75.0)	23 (39.0)	2.952	6	.82	2 (100.0)	2 (33.3)	0	22 (40.0)	0	13.530	12	.33	26 (41.3)
20 000 to <40 000	0	16 (27.1)				0	0	0	16 (29.1)	0				16 (25.4)
40 000 to <60 000	1 (25.0)	11 (18.6)				0	2 (33.3)	0	10 (18.2)	0				12 (19.0)
60 000 to <80 000	0	3 (5.1)				0	0	0	3 (5.5)	0				3 (4.8)
80 000 to <100 000	0	2 (3.4)				0	1 (16.7)	0	1 (1.8)	0				2 (3.2)
100 000 to <120 000	0	2 (3.4)				0	1 (16.7)	0	1 (1.8)	0				2 (3.2)
120 000 to <140 000	0	2 (3.4)				0	0	0	2 (3.6)	0				2 (3.2)
Total cost, $	80 000	2 130 000				20 000	320 000	0	1 870 000	0				13 859 000

Abbreviation: ART, assisted reproductive technology.

^a^ Total numbers may vary due to item non-response. Only participants who reported their age were included.

^b^ Only asked of participants who have used assisted reproductive technology.

## Discussion

This survey study found that medical students and physicians incur substantial costs to use ART, with some spending over $140 000. The time during which physicians often consider building their families intersects with the earliest stages of their careers, a time when these amounts may be prohibitive.^5^ This is particularly problematic for individuals who are single or in same-sex partnerships and have few family building options beyond ART, adoption, or gestational carriers. Stigma associated with childbearing among trainees is also a major deterrent to family building, with many expressing concerns about faculty and other residents’ views and the lack of availability of and transparency around policies and resources to support building families.^3,6^ Limitations of this study include sampling bias inherent to social media recruitment, cross-sectional data, binary age grouping likely underestimating infertility experienced by older participants, and inability to directly compare our data with the general population.

Our results highlight why we must better support trainees building their families before their fertility has declined through appropriate parental leave policies, on-site free or low-cost childcare, and insurance coverage for all the ways in which people build families.^3^ Health care workers who want children should not have to sacrifice building families for the sake of their careers, and it is the responsibility of health care organizations to prevent that from happening.

## Notes

Supplement.
Data Sharing Statement
