# Supplementary material for: Costs of Family Building for Physicians and Medical Students
Source: JAMA Netw Open. 2025 Jan 29;8(1):e2457287. doi: 10.1001/jamanetworkopen.2024.57287 (PMC11780473; doi:10.1001/jamanetworkopen.2024.57287)
Supplement: Supplement. — Data Sharing Statement [file jamanetwopen-e2457287-s001.pdf]

Data Sharing Statement

Levy. Costs of Family Building for Physicians and Medical Students. *JAMA Netw Open*. Published January 29, 2025. doi:10.1001/jamanetworkopen.2024.57287

Data
Data available: Data available upon request.
